# A low-carbon supply chain pricing mechanism considering CSR under carbon cap-and-trade policy

**DOI:** 10.1371/journal.pone.0311913

**Published:** 2024-10-25

**Authors:** Hao Zou, Jin Xiao, Yuanyuan Lou, Dan Liao, Huwei Deng, Jiehui Jiang

**Affiliations:** School of Business Administration, Hunan University of Finance and Economics, Changsha, China; National Textile University, PAKISTAN

## Abstract

In the context of environmental deterioration and people’s growing environmental protection awareness, governments or regions have put forward corresponding carbon emission reduction policies. Among them, the carbon trading mechanism, as an effective means to promote enterprises to implement emission reduction measures, plays a crucial role in regulating enterprise behavior and promoting social sustainable development. Since various industries and sectors support each other in social and economic development, it is more reasonable to study the carbon emission reduction optimization decisions of society and enterprises from the perspective of the supply chain. To achieve the carbon reduction target of the supply chain system, manufacturing enterprises usually need to incur additional costs to invest in emission reduction technologies, and retail enterprises also need to conduct low-carbon publicity to increase product market share. On one hand, considering the impact of the government’s emission reduction constraints and consumers’ low-carbon preferences, manufacturers will take corporate social responsibility (CSR) into consideration to enhance product competitiveness. On the other hand, smaller retailers are more concerned about being treated fairly than about their own profits due to the extra cost of low-carbon advertising. In this paper, considering the background of carbon trading, the manufacturer’s CSR and retailer’s fairness concern behavior are introduced into the decision-making process of the low-carbon supply chain (LCSC), and the relevant emission reduction decision-making model is constructed by using Stackelberg game theory and backward derivation method. Through comparative analysis of relevant parameters, members’ profits and utilities, this paper focuses on the influence of CSR and fairness concerns on system decision-making. The results show that the optimal way for LCSC decision-making is to cooperate with fair-concerned retailers and manufacturers with CSR. When manufacturers consider social responsibility within a certain range and retailers bear part of the cost of social responsibility as followers, it can not only effectively improve the emission reduction level of the supply chain and the profits of each entity, but also help to increase the enthusiasm of each entity for carbon emission reduction and the overall social welfare.

## 1. Introduction

With the prosperity of economy and the continuous development of science and technology, a large amount of greenhouse gases are emitted in the manufacturing process, which seriously affects the stability of global climate and environmental ecological security [1]. In order to mitigate the greenhouse effect and curb the continued deterioration of the environment, countries or regions have successively issued strict international environmental protection laws and relevant laws and regulations, such as Germany’s "Closed Circulation of Materials and Waste Management Law" [2], Japan’s "Kyoto Protocol"[3,4], China’s “Carbon Emission Trading Management Measures (Trial)” [5]. Although these relevant environmental protection laws and regulations have played a significant role in increasing the low-carbon and environmental awareness of governments, enterprises and consumers around the world [6], global greenhouse gas emissions have not been significantly reduced [7]. According to relevant studies, global greenhouse gas emissions continue to increase, and the problem of global temperature rise will be further aggravated in the future [8]. At this critical moment, "how to vigorously reduce emissions" has become the focus of global debate. In response to the relevant appeals and calls of the United Nations, countries around the world are trying to reduce greenhouse gas emissions by various means, implement environmental protection laws such as the Paris Agreement, and alleviate environmental problems [9]. For example, countries around the world have introduced a series of carbon emission reduction policies such as carbon tax and carbon tariff, and proposed carbon neutrality targets to achieve net zero emissions [10], increased investment in carbon emission reduction technologies and levied environmental taxes, especially energy taxes [11]. However, compared with the carbon tax policy, the procedure of carbon cap and carbon trading mechanism is simple and flexible, and directly points to carbon emissions, and the emission reduction effect is more obvious. Many countries and regions have established their own carbon trading markets. For example, the European Union promotes the Emissions Trading System [12], Japan introduces an emissions trading system [13], South Korea implements a carbon trading market [14], and China implements carbon trading policies [15], etc.Meanwhile, carbon credits, as exchangeable products in the market, can attract more banks, funds and enterprises to participate and improve the efficiency of resource allocation [16,17]. Therefore, countries around the world will pay more attention to the impact of carbon trading on decision-making when making supply chain decisions.

Under the influence of government regulation, increasing competition pressure in the same industry, and increasing entrepreneur awareness, more and more enterprises begin to pay attention to the role of corporate social responsibility (CSR) [18]. Some studies have shown that an enterprise’s active commitment to social responsibility can enhance the relevant image of the enterprise and, to a certain extent, satisfy the low-carbon preference of consumers [19], improve the competitiveness and recognition of the enterprise in the market [20], and has an important role in increasing the relevant profits of enterprises and enhancing the overall social value of enterprises. At present, some enterprises have taken corresponding measures to actively undertake CSR. For example, Procter & Gamble promoted the "Shiksha" project in India [21], Nike donated in-kind during the pandemic [22], Toyota Motor implemented a green product design strategy [23], the Carlsberg beer Group hired experts to propose solutions to reduce pollution in the production process [24]. A series of initiatives of these enterprises reflect the increasing importance of CSR in the decision-making of supply chain. However, in real life, it should be noted that with the gradual promotion of "low-carbon" goals and the continuous development of carbon trading market, some manufacturing enterprises with large pollution emissions will adopt advanced technology treatment processes to meet carbon emission standards while actively undertaking CSR [25]. To a certain extent, this method will prolong the production and delivery cycle of products, increase the production cost of the enterprise [26], and increase the operating pressure and capital occupation of the enterprise virtually. At the same time, due to the rising production cost of enterprises, the market price of low-carbon products is higher than that of ordinary products to some extent, and some consumers will choose to buy cheaper ordinary products, which is not conducive to the market promotion and sales of low-carbon products [27]. Therefore, in the manufacturer-led LCSC, manufacturers need to assume greater social responsibility, but in this process, the business conditions of enterprises need to be considered, and the comprehensive impact of CSR on corporate reputation, society, environment and other aspects should be paid attention to.

In the context of carbon trading, enterprises will not only consider maximizing their own interests, but also consider communicating and developing the cooperation relationship between the upstream and downstream of the supply chain, so as to pay attention to the fair concern behavior of the whole supply chain subject [28]. As a common behavioral preference of decision makers,fairness concerns will not only affect the emission reduction decisions of supply chains [29], but also directly affect the operations and decisions of enterprises [30]. For example, Tingyi unilaterally raised wholesale prices without considering fair behavior, leading to the failure of cooperation with Guangzhou Friendship Group; Lanza Group terminated its cooperation with Walmart because its interests were unfairly treated [31]. In the LCSC, the relationship between the members of the supply chain is more complex, and the problem that retailers pay attention to the fair distribution of income because of the low-carbon publicity investment is more prominent. At this time, CSR and fairness concerns in the context of carbon trading are not only conducive to reducing carbon emissions, but also to achieving sustainable development among supply chain members. Therefore, in the context of carbon trading, it is particularly important to consider the fair concern behavior of the LCSC members.

To sum up, in the case of climate deterioration and ecological security problems caused by greenhouse gas emissions, countries around the world have formulated and introduced a series of carbon emission reduction policies to reduce carbon emissions. At the same time, under the pressure of low-carbon goals and stimulated by consumers’ preference for green consumption, enterprises will take more consideration of manufacturers’ CSR and retailers’ fairness concerns when making supply chain decisions from the perspective of overall profits. However, there is little existing literature on the joint impact of CSR of manufacturers and fair concerns of retailers on LCSC in the context of carbon trading. Based on the above reasons, this paper constructs a CSR model under fairness concerns in the context of carbon trading by Stackelberg game theory, and uses backward derivation to study the impact of manufacturers’ CSR and retailers’ fairness concerns on LCSC decision-making, and further puts forward suggestions for LCSC operational decision-making. In this study, we tried to address the following questions: (1) In the context of carbon trading, what is the impact of consumers’ low-carbon preference and low-carbon cost coefficient on LCSC decision-making? (2) What are the differences between retailer fairness concerns and fairness neutrality in LCSC decision-making? (3) How do different levels of manufacturer CSR affect LCSC decisions? (4) How can CSR and fairness concerns be designed to improve the overall profits and utility of the LCSC?

In order to solve the above problems, this paper considers the carbon trading background and constructs a two-level LCSC composed of a manufacturer, a retailer and a consumer. Among them, the manufacturer is the Stackelberg leader, the retailer is the follower who pays attention to the manufacturer’s CSR, and the consumer has the consumer surplus. The purpose of this paper is to explore the impact of the combination of manufacturer CSR and retailer fairness concerns on LCSC decision-making in the context of carbon trading. The main contributions of this paper are as follows. (1) Different from existing studies, in the manufacturer-led two-level LCSC, we not only consider the impact of manufacturer CSR and retailer’s fair concern behavior on LCSC decision-making, but also take into account the background of carbon trading mechanism. This paper explicitly explores the impact of CSR and fairness concerns on two-level LCSC decision-making in the context of carbon trading mechanisms. (2) Based on Stackelberg game theory, this paper constructs the decision models of manufacturers’ CSR and retailers’ fairness concerns under different models, and obtains the optimal values of emission reduction rate, wholesale price, profit and social welfare of LCSC system by using backward derivation method. (3) Through numerical simulation and comparative analysis, this paper obtains some interesting management implications of manufacturer CSR and retailer fair concern behavior for LCSC decision-making. After the manufacturers invest in CSR, although the sales profit will be slightly reduced, it can improve their own utility and the overall LCSC profit to a large extent. Although the fair concern behavior of retailers contributes to the overall development of LCSC to a certain extent, excessive investment will seriously damage the profits of manufacturers and is not conducive to the sustainable development of LCSC.

The rest of the paper is structured as follows: Section 2 provides a comprehensive literature review based on carbon trading, CSR, and fairness concerns. Section 3 describes the problem and makes basic assumptions, and further constructs the decision model of different situations. Section 4 compares and analyzes each index in the LCSC. Section 5 is the numerical analysis and the results are discussed. Section 6 summarizes the research of this paper, and puts forward relevant suggestions for LCSC decision-making based on the above analysis.

## 2. Literature review

The literature related to this paper can be divided into three aspects. The first is the study of LCSC in the context of carbon trading, the second is the study of fairness concerns in the context of carbon trading, and the last is the study considering the impact of CSR on LCSC. This article will focus on reviewing the literature that is particularly relevant to this topic.

### 2.1 Research on LCSC management in the context of carbon trading

In recent years, sustainable supply chain management has attracted increasing scholarly attention [32]. Within this field, research on low-carbon supply chain operation management primarily focuses on consumers’ low-carbon preferences and investment in carbon emission reduction technologies. Regarding the study of consumers’ low-carbon preference in operation management, Li et al. (2018) [33] examined enterprises’ decision-making strategies for low-carbon initiatives and social welfare considering varying consumer preferences. Kang et al. (2019) [34] further explored the customization of low-carbon products by retailers and manufacturers’ financing, delving into the decision-making process behind supply chain enterprises’ low-carbon strategy. Additionally, some scholars have investigated the decision-making process for supply chain emission reduction based on uncertain levels of low carbon preference. For instance, Xu et al. (2023) [35] studied manufacturers’ emission reduction investment strategies considering random low-carbon preferences. As for research on investment in carbon emission reduction technology, Luo et al. (2016) [36] discussed decision-making issues within a competitive and cooperative game between two manufacturers with different emission reduction efficiencies; Sun et al.(2020) [37] explored the carbon emission transfer strategy of the supply chain while considering the impact of lagging emission reduction technology; Chen et al.(2022) [38]studied profit distribution and social welfare performance among supply chain members while taking into account both manufacturer competition and low-carbon technology transfer.

In comparison to a carbon tax, the carbon trading policy offers greater flexibility and convenience, enhances resource utilization, and enjoys broader acceptance among supply chain enterprises. In recent years, numerous scholars have examined low-carbon supply chain decision-making within the framework of carbon trading from various perspectives. Relevant studies mainly include pricing of low-carbon supply chain members [39,40], production decisions [41], recycling [42,43], and emission reduction decisions [44,45], etc. Furthermore, considering that carbon emission reduction investment will incur additional costs, some scholars have further incorporated subsidy policies and financing strategies into the decision-making process of low-carbon supply chains. Zhang and Zhang (2022) [46] took the government’s green subsidy strategy into account and investigated the influence of the green subsidy coefficient on the stability of the low-carbon supply chain system. Cong et al. (2020) [47] considered the green financial subsidy mechanism and examined the optimal decision-making issue of the low-carbon supply chain under capital constraints.

### 2.2 The influence of fairness concern behavior on LCSC decision-making

The above-mentioned literature on the operation and management of low-carbon supply chain are all based on the behavioral neutral situation, while in real life, decision makers, as bounded rational economic men, will consider whether the income distribution is fair while maximizing their own profits. Some scholars have introduced fairness concern behavior into service supply chain [48], tourism supply chain [49], fresh cold supply chain supply chain [50], etc.However, in the low-carbon supply chain, manufacturers have to invest in emission reduction technologies due to the implementation of national carbon policies and regulations, and retailers have to face an uncertain market environment due to the low-carbon preferences of consumers, making both of them very concerned about whether they are treated fairly. At present, some scholars have separately considered the fairness concerns of different members, and studied the pricing and emission reduction decision-making problems of low-carbon supply chain.

On the one hand, scholars have considered the fairness concern behavior of retailers and studied the emission reduction and coordination decision-making issues of the low-carbon supply chain. Zhou et al. (2016) [51] built a joint contract based on advertising cooperation and cost sharing mechanism to realize the coordination of low-carbon supply chain, considering the fairness concerns of retailers. Aiming at different demand markets, Wu and Xia (2023) [52] explored the impact of retailer’s fair concern behavior on the balanced decision-making of low-carbon supply chain. Sun and Zhong (2023) [53] further considered the level of green marketing efforts of retailers and studied the impact of fairness concerns on emission reduction decisions in supply chain systems.On the other hand, some scholars have introduced manufacturers’ fairness concerns into the decision-making process of low-carbon supply chain. Han et al. (2020) [54] introduced the government carbon subsidy mechanism and manufacturers’ fair concern behavior into the e-commerce supply chain, and studied the emission reduction decision-making and coordination of the supply chain system. Shi (2022) [55] studied the influence of manufacturers’ fair concern behavior on the profit distribution of supply chain members based on carbon tax policy.

In addition, Li et al. (2019) [56] studied the decision-making problem of low-carbon dual-channel supply chain considering the fairness concern behavior of both retailers and manufacturers. Song et al. (2024) [57] further considered consumers’ fairness concern behavior and studied the impact of product utility gap on the purchase intention of consumers with low-carbon preferences. The above literature on fairness concerns does not focus on the issue of corporate social responsibility in low-carbon supply chains. In contrast, we focus on the impact of carbon trading policies and corporate social responsibility on supply chain decisions.

### 2.3 The impact of CSR behavior on LCSC decision-making

In the current fierce market competition environment, stakeholders expect more enterprises to fulfill CSR, so CSR has gradually become a favorable tool to improve the core competitiveness of enterprises. CSR can not only promote a good corporate image, but also bring greater value to consumers or the society as a whole [58]. Some scholars have introduced CSR behavior into the decision-making process of supply chain operations. For example, Valdez-Juarez et al. (2018) [59] studied the impact of corporate social responsibility and supply chain management on corporate performance. Raza (2018) [60] studied supply chain coordination decision-making based on corporate social responsibility. Johari et al. (2019) [61] studied the pricing decision-making process of manufacturers considering CSR behavior. Chan et al. (2020) [62] and Hafezalkotob et al. (2023) [63] explored CSR issues in fashion supply chains and smart supply chains respectively.

Compared with the traditional supply chain, the related enterprises of low-carbon supply chain need to assume greater social responsibility. This has also aroused widespread concern among scholars. Wang et al. (2023) [64] discussed the carbon emission reduction decision-making of manufacturers in a sustainable supply chain, considering the CSR behavior of retailers. Wu and Li (2022) [27] discussed the optimal decision-making of technological innovation level of low-carbon supply chain by considering three CSR commitment models. He et al. (2020) [65] introduced CSR behavior into low-carbon service supply chain and studied the design of cost sharing contract. Based on the background of carbon emission tax, Modak and Kelle (2021) [21] studied the issue of corporate social responsibility in closed-loop supply chain. The above literature on corporate social responsibility does not address the fairness concern behavior of supply chain members. However, our research focuses on the impact of carbon trading mechanisms and fairness concerns on low-carbon supply chain decisions.

Some scholars have studied the operational decision-making of low-carbon supply chain based on fairness concern behavior, but few have incorporated CSR behavior and fairness concern characteristics into the decision-making process of low-carbon supply chain. In the literature most closely related to this paper, Wang et al. (2021) [5] and Chen et al. (2023) [66] studied the problem of balanced decision-making of closed-loop supply chain considering corporate social responsibility and fairness concern behavior. In contrast, our research is based on a low carbon supply chain perspective and takes into account carbon cap-and-trade mechanisms.

## 3. Model construction and solution

### 3.1 Symbol explanation and basic assumptions

This paper constructs a LCSC composed of a manufacturer, a retailer and a consumer. In the context of the free allocation of certain carbon quotas by the government, manufacturers in the supply chain invest in emission reduction technologies to reduce carbon emissions, and ensure a certain level of carbon emission reduction by improving corporate social responsibility. Retailers exhibit some fairness concerns by investing in low-carbon advertising and expect to sell their products through different channels. Based on the preceding description, the related parameters are shown as follows.

*c*: Manufacturer’s cost of production;
*ω*: Manufacturer’s wholesale price per unit product;
*P*: Retailers wholesale price per unit of product;
*t*: Unit carbon emission trading price;
*e*: Manufacturer’s carbon emissions per initial unit of product;
*β*: Manufacturer’s emission reduction level;
*Q*: Market demand;
*G*: Total carbon credits allocated to manufacturers by the government;
*M*: Carbon reduction cost factor;
*ϕ*: Fairness concern coefficient;
*CS*: Consumer surplus;
*λ*: Low carbon preference coefficient of consumers;
*b*: Sales price sensitivity coefficient;
*θ*(0<*θ*<1): CSR behavior coefficient of manufacturers;
*π*(*G*): Manufacturers’ earnings from carbon trading;
*π*_*m*_: Manufacturer’s profit;
*π*_*r*_: Retailer profit;
*π*_*c*_: Total supply chain profit;
*U*_*m*_: Manufacturer utility function;
*U*_*r*_: Retailer utility function;
*W*: Social welfare;
*N*: Manufacturers without CSR;
*C*: Manufacturers with CSR;
*A*: Retailer fair neutral situation;
*F*: Retailer fair concern situation.

In order to facilitate modeling and solving, this paper makes basic assumptions.

Hypothesis 1: In order to meet the government’s carbon emission regulation and consumers’ low-carbon preference demand, manufacturers will invest in emission reduction technologies. Referring to the study of Zhou et al. (2016) [51], the manufacturer’s emission reduction cost is described as a function related to emission reduction cost coefficient and emission reduction level: *Mβ*^2^/2. In addition, according to the study of Xu et al. (2023) [25], the product market demand function is described as a function related to the sales price and the low carbon preference coefficient of consumers: *Q* = *a*−*bp*+*λβ*.
Hypothesis 2: With reference to Cheng et al. (2023) [10], the consumer surplus is described as the difference between the highest price that consumers can accept and the actual price: $CS=\int_{p}^{p_{\max}}QdP\int_{\frac{a+\lambda \beta -Q}{b}}^{\frac{a+\lambda \beta }{b}}\left(a-bP+\lambda \beta \right)dP=\frac{{\left(a-bP+\lambda \beta \right)}^{2}}{2b}$. We further describe the utility of the manufacturer as a function of its own profit related to the consumer surplus. The specific expression is as follows: $U_{m}=\pi_{m}+\theta *\mathrm{CS}=\pi_{m}+\theta {\left(a-bP+\lambda \beta \right)}^{2}/2b,(0<\theta <1)$. When *θ* = 0, the LCSC pursues the maximum profit; When *θ* = 1, the LCSC pursues the maximization of social welfare.
Hypothesis 3: According to Zhang et al. (2020) [45], the social welfare is described as the sum of manufacturer’s profit, retailer’s profit and consumer surplus, with specific expression as *W* = *π*_*m*_+*π*_*r*_+*CS*.

### 3.2 Model construction under different circumstances

In order to facilitate comparative analysis of the influence of CSR and fairness concern behaviors on pricing and emission reduction decisions of LCSC, four supply chain decision-making situations are discussed here. (1) Behavior decisions that do not consider CSR and fairness concerns. Manufacturers do not consider CSR behavior, and retailers make fair and neutral decisions. (2) Only consider fairness concerns in decision-making. Retailers use the absolutely fair model of income distribution to describe utility losses, while manufacturers make fair neutral decisions. (3) Only CSR behavior decisions are considered. Manufacturers focus on the optimal utility after CSR input, while retailers make fair and neutral decisions. (4) Consider both CSR and fairness concerns in decision-making. Manufacturers make CSR inputs and retailers make fair concern decisions.

#### 3.2.1 Situations where CSR and fairness concerns are not taken into account

In this model, maximizing profit is the decision goal of manufacturers and retailers. The manufacturer determines the wholesale price of the product a and the low carbon level of the product b, and aims to maximize its own profit. Retailers respond to the actions of manufacturers and determine the price of products with the goal of maximizing their own profits. The profit function of manufacturer, retailer and supply chain system can be expressed as:

$$\pi_{m}^{NA}=(\omega -c)Q+t[G-e(1-\beta )Q]-M\beta^{2}/2$$ (1)

$$\pi_{r}^{NA}=(P-\omega )Q$$ (2)

$$\pi_{c}^{NA}=(P-c)Q+t[G-e(1-\beta )Q]-M\beta^{2}/2$$ (3)

The method of backward derivation is adopted. Since $\partial^{2}\pi_{r}^{NA}/\partial p^{2}=-2b$ and $\partial \pi_{r}^{NA}/\partial P=0$, the reaction function $P_{1}^{NA}=(\omega b+\lambda \beta +\alpha )/2b$ of retail price with respect to wholesale price and carbon emission reduction rate can be obtained, which is further substituted into $\pi_{m}^{NA}=(\omega -c)Q+t[G-e(1-\beta )Q]-M\beta^{2}/2$, and the Hessian matrix $H_{1}(\omega ,\beta )=[\begin{array}{cc} -b & \left(\lambda -teb\right)/2\\ \left(\lambda -teb\right)/2 & te\lambda -M \end{array}]$ with respect to wholesale price *ω* and carbon emission reduction rate *β*. Because first-order sequential principal subformula *D*_1_ = −*b*<0, *H*_1_(*ω*,*β*) is a negative definite matrix when second-order sequential principal subformula *D*_2_ = [4*bM*−(*λ*+*teb*)^2^]/4>0. Therefore, by combining $\partial \pi_{m}^{NA}/\partial \omega =0$ and $\partial \pi_{m}^{NA}/\partial \beta =0$, the optimal wholesale price, carbon emission reduction level, retail price and optimal order quantity can be obtained as follows:

$$\omega^{NA}=\frac{2M(bet+bc+a)-(bet+\lambda )(aet+\lambda et+\lambda c)}{4Mb-{\left(bet+\lambda \right)}^{2}}$$ (4)

$$\beta^{NA}=\frac{\left(a-bet-bc)(bet+\lambda \right)}{4Mb-{\left(bet+\lambda \right)}^{2}}$$ (5)

$$P^{NA}=\frac{M(bet+bc+3a)-(bet+\lambda )(aet+et\lambda +c\lambda )}{4Mb-{\left(bet+\lambda \right)}^{2}}$$ (6)

$$Q^{NA}=\frac{bM(a-bet-bc)}{4Mb-{\left(bet+\lambda \right)}^{2}}$$ (7)

Further, the manufacturer’s profit, retailer’s profit, overall supply chain profit, consumer surplus and social welfare can be obtained as follows:

$$\pi_{m}^{NA}=\frac{M{\left(a-bet-bc\right)}^{2}}{2[4Mb-{\left(bet+\lambda \right)}^{2}]}+tG$$ (8)

$$\pi_{r}^{NA}=\frac{bM^{2}{\left(a-bet-bc\right)}^{2}}{{\left[4Mb-{\left(bet+\lambda \right)}^{2}\right]}^{2}}$$ (9)

$$\pi_{c}^{NA}=\frac{M{\left(a-bet-bc\right)}^{2}[6bM-{\left(\lambda +teb\right)}^{2}]}{2{\left[4Mb-{\left(bet+\lambda \right)}^{2}\right]}^{2}}+tG$$ (10)

$$CS^{NA}=\frac{M^{2}b{\left(a-bet-bc\right)}^{2}}{2{\left[4Mb-{\left(bet+\lambda \right)}^{2}\right]}^{2}}$$ (11)

$$W^{NM}=\frac{M{\left(a-bet-bc\right)}^{2}[6bM+Mb^{2}-{\left(\lambda +teb\right)}^{2}]}{2{\left[4Mb-{\left(bet+\lambda \right)}^{2}\right]}^{2}}+tG$$ (12)

On the other hand, when the members of the LCSC make centralized decisions, the Hessian matrix $H_{2}(P,\beta )=[\begin{array}{cc} -2b & \lambda -teb\\ \lambda -teb & 2te\lambda -M \end{array}]$ can be obtained for $\pi_{c}^{NA}$ with respect to the selling price *P* and the carbon emission reduction rate *β*. Because the first order principal subformula *D*_1_ = −2*b*<0, then when the second order principal subformula *D*_2_ = 2*bM*−(*λ*+*teb*)^2^>0, *H*_2_(*P*,*β*) is a negative definite matrix, and further simultaneous $\partial \pi_{c}^{NA}/\partial P=0$ and $\partial \pi_{c}^{NA}/\partial \beta =0$ can obtain the optimal parameter values under the centralized decision. Since this paper mainly compares the influence of CSR behavior and fairness concern behavior on decision-making of LCSC members, and also facilitates the comparison of different parameters, only 2*bM*−(*λ*+*teb*)^2^>0 is taken as an important condition for subsequent derivation and analysis.

#### 3.2.2 Consider only fairness concerns

In this model, the manufacturer makes the decision to maximize its own profit, while the retailer makes the decision to maximize its own utility considering the fairness factor. Referring to Li et al. (2019) [56], FS model is used to describe the retailer’s fair concern behavior, then the manufacturer’s profit function and retailer’s utility function are as follows:

$$\pi_{m}^{NF}=(\omega -c)Q+t[G-e(1-\beta )Q]-M\beta^{2}/2$$ (13)

$$U_{r}^{NF}=(P-\omega )(a-bP+\lambda P)-\varphi \{(2\omega -c-p)Q+t[G-e(1-\beta )Q]-M\beta^{2}/2\}$$ (14)

Similar to 3.2.1, this study adopts the backward derivation method. When 4*Mb*(2*ϕ*+1)−(*teb*+*λ*)^2^(*ϕ*+1)>0, the optimal wholesale price, carbon reduction level, retail price and optimal order quantity under the retailer’s fairness concern can be obtained as:

$$\omega^{NF}=\frac{2M[b(3\phi +1)(teb+bc)+a(\phi +1)]-(teb+\lambda )(\phi +1)(aet+te\lambda +c\lambda )}{4Mb(2\phi +1)-{\left(teb+\lambda \right)}^{2}(\phi +1)}$$ (15)

$$\beta^{NF}=\frac{\left(teb+\lambda )(\phi +1)(a-teb-bc\right)}{4Mb(2\phi +1)-{\left(teb+\lambda \right)}^{2}(\phi +1)}$$ (16)

$$P^{NF}=\frac{M(2\phi +1)(teb+bc+3a)-(teb+\lambda )(\phi +1)(aet+te\lambda +c\lambda )}{4Mb(2\phi +1)-{\left(teb+\lambda \right)}^{2}(\phi +1)}$$ (17)

$$Q^{NF}=\frac{Mb(2\phi +1)(a-teb-bc)}{4Mb(2\phi +1)-{\left(teb+\lambda \right)}^{2}(\phi +1)}$$ (18)

Further, the manufacturer’s profit, retailer’s profit, overall supply chain profit, retailer’s utility, consumer surplus and social welfare can be obtained as follows:

$$\pi_{m}^{NF}=\frac{M{\left(a-teb-bc\right)}^{2}(\phi +1)}{2[4Mb(2\phi +1)-{\left(teb+\lambda \right)}^{2}(\phi +1)]}+Gt$$ (19)

$$\pi_{r}^{NF}=\frac{M^{2}b{\left(a-teb-bc\right)}^{2}(2\varphi +1)(4\varphi +1)}{{\left[4Mb(2\varphi +1)-{\left(teb+\lambda \right)}^{2}(\varphi +1)\right]}^{2}}$$ (20)

$$\pi_{c}^{NF}=\frac{M{\left(a-teb-bc\right)}^{2}[6Mb{\left(2\varphi +1\right)}^{2}-{\left(\varphi +1\right)}^{2}{\left(teb+\lambda \right)}^{2}]}{2{\left[4Mb(2\varphi +1)-{\left(teb+\lambda \right)}^{2}(\varphi +1)\right]}^{2}}+Gt$$ (21)

$$U_{r}^{NF}=\frac{M(1+\varphi ){\left(a-teb-bc\right)}^{2}[2Mb{\left(2\varphi +1\right)}^{2}+\varphi {\left(teb+\lambda \right)}^{2}]}{2{\left[4Mb(2\varphi +1)-{\left(teb+\lambda \right)}^{2}(\varphi +1)\right]}^{2}}+\varphi Gt$$ (22)

$$CS^{NF}=\frac{b}{2}{\left[\frac{M(2\phi +1)(a-teb-bc)}{4Mb(2\phi +1)-{\left(teb+\lambda \right)}^{2}(\phi +1)}\right]}^{2}$$ (23)

$$W^{NF}=\frac{M{\left(a-teb-bc\right)}^{2}[7Mb{\left(2\phi +1\right)}^{2}-{\left(\phi +1\right)}^{2}{\left(teb+\lambda \right)}^{2}]}{2{\left[4Mb(2\phi +1)-{\left(teb+\lambda \right)}^{2}(\phi +1)\right]}^{2}}+Gt$$ (24)

#### 3.2.3 Consider only the CSR scenario

In this model, manufacturers pay attention to CSR behavior, and their concern for social responsibility constitutes a new utility function. At this time, the manufacturer’s objective function is not simply to pursue the maximum profit, but to pay more attention to the optimal utility after the introduction of CSR. Retailers make decisions based on fair and neutral behavior to maximize their own profits. As the dominant player in the game, the manufacturer first determines the wholesale price *ω* and carbon reduction level *β* of the product according to its own utility maximization, and then the retailer, as a follower, determines the sales price *P* of the product. With reference to Cheng et al. (2023) [10], the utility function of the manufacturer and the profit function of the retailer are as follows:

$$U_{m}=(\omega -c)Q+t[G-e(1-\beta )Q]-M\beta^{2}/2+\theta Q^{2}/2b$$ (25)

$$\pi_{r}=(P-\omega )Q$$ (26)

Similarly, when *Mb*(4−*θ*)−(*bet*+*λ*)^2^>0, the optimal wholesale price, carbon emission reduction level, retail price and optimal order quantity of the manufacturer with CSR behavior can be obtained as follows:

$$\omega^{CA}=\frac{\left(teb+\lambda )(aet+te\lambda +\lambda c)+M(a\theta -2teb-2cb-2a\right)}{{\left(bet+\lambda \right)}^{2}+Mb(\theta -4)}$$ (27)

$$\beta^{CA}=-\frac{\left(teb+\lambda )(a-teb-bc\right)}{{\left(bet+\lambda \right)}^{2}+Mb(\theta -4)}$$ (28)

$$P^{CA}=\frac{\left(teb+\lambda )(aet+te\lambda +\lambda c)+M(a\theta -teb-bc-3a\right)}{{\left(bet+\lambda \right)}^{2}+Mb(\theta -4)}$$ (29)

$$Q^{CA}=-\frac{Mb(a-teb-bc)}{{\left(bet+\lambda \right)}^{2}+Mb(\theta -4)}$$ (30)

Further, the manufacturer’s profit, retailer’s profit, overall supply chain profit, manufacturer’s utility, consumer surplus and social welfare can be obtained as follows:

$$\pi_{m}^{CA}=\frac{-M{\left(a-teb-bc\right)}^{2}[{\left(teb+\lambda \right)}^{2}+2Mb(\theta -2)]}{2{\left[{\left(bet+\lambda \right)}^{2}+Mb{\left(\theta -4\right)}^{2}\right]}^{2}}+tG$$ (31)

$$\pi_{r}^{CA}=\frac{M^{2}b{\left(a-teb-bc\right)}^{2}}{{\left[{\left(bet+\lambda \right)}^{2}+Mb(\theta -4)\right]}^{2}}$$ (32)

$$\pi_{c}^{CA}=\frac{M{\left(a-teb-bc\right)}^{2}[2Mb(3-\theta )-{\left(teb+\lambda \right)}^{2}]}{2{\left[{\left(bet+\lambda \right)}^{2}+Mb(\theta -4)\right]}^{2}}+tG$$ (33)

$$U_{m}^{CA}=\frac{-M{\left(a-teb-bc\right)}^{2}}{2[{\left(bet+\lambda \right)}^{2}+Mb(\theta -4)]}+tG$$ (34)

$$CS^{CA}=\frac{b}{2}{\left[\frac{M(a-teb-bc)}{{\left(teb+\lambda \right)}^{2}+Mb(\theta -4)}\right]}^{2}$$ (35)

$$W^{CA}=\frac{M{\left(a-teb-bc\right)}^{2}[Mb(7-2\theta )-{\left(teb+\lambda \right)}^{2}]}{2{\left[{\left(teb+\lambda \right)}^{2}+Mb(\theta -4)\right]}^{2}}$$ (36)

#### 3.2.4 Consider both CSR and fairness concerns

It is assumed that manufacturers consider the impact of corporate social responsibility when investing in emission-reduction technologies, while retailers exhibit certain fair preference behaviors due to low-carbon promotion. Combined with 3.2.2 and 3.2.3, the utility functions for manufacturers and retailers can be expressed as:

$$U_{m}=(\omega -c)Q+t[G-e(1-\beta )Q]-M\beta^{2}/2+\theta Q^{2}/2b$$ (37)

$$U_{r}=(p-\omega )(a-bP+\lambda P)-\varphi \{(2\omega -c-p)Q+t[G-e(1-\beta )Q]-M\beta^{2}/2\}$$ (38)

Similarly, when ${\left(teb+\lambda \right)}^{2}{\left(\varphi +1\right)}^{2}+Mb(2\varphi +1)(2\varphi \theta -4\varphi +\theta -4)<0$, the optimal wholesale price, carbon emission reduction level, retail price and optimal order quantity under the condition that the manufacturer has CSR and the retailer shows fair concern can be obtained as follows:

$$\omega^{CF}=\frac{\left(ate+te\lambda +c\lambda )(teb+\lambda ){\left(\phi +1\right)}^{2}+Ma(\phi +1)(2\phi \theta -2\phi +\theta -2\right)}{{\left(teb+\lambda \right)}^{2}{\left(\phi +1\right)}^{2}+Mb(2\phi +1)(2\phi \theta -4\phi +\theta -4)}$$

$$+\frac{Mb(et+c)(2\phi^{2}\theta -6\phi^{2}+\phi \theta -8\phi -2)}{{\left(teb+\lambda \right)}^{2}{\left(\phi +1\right)}^{2}+Mb(2\phi +1)(2\phi \theta -4\phi +\theta -4)}$$ (39)

$$\beta^{CF}=\frac{{\left(\phi +1\right)}^{2}(ebt+\lambda )(ebt+bc-a)}{{\left(teb+\lambda \right)}^{2}{\left(\phi +1\right)}^{2}+Mb(2\phi +1)(2\phi \theta -4\phi +\theta -4)}$$ (40)

$$P^{CF}=\frac{M(2\phi +1)[a\theta (2\phi +1)-(\phi +1)(teb+bc+3a)]+(teb+\lambda ){\left(\phi +1\right)}^{2}(aet+te\lambda +c\lambda )}{{\left(teb+\lambda \right)}^{2}{\left(\phi +1\right)}^{2}+Mb(2\phi +1)(2\phi \theta -4\phi +\theta -4)}$$ (41)

$$Q^{CF}=\frac{Mb(2\phi +1)(\phi +1)(ebt+bc-a)}{{\left(teb+\lambda \right)}^{2}{\left(\phi +1\right)}^{2}+Mb(2\phi +1)(2\phi \theta -4\phi +\theta -4)}$$ (42)

Further, the manufacturer’s profit, retailer’s profit, overall supply chain profit, manufacturer’s utility, retailer’s utility, consumer surplus and social welfare can be obtained as follows:

$$\pi_{m}^{CF}=\frac{-M{\left(\phi +1\right)}^{2}(2\phi +1){\left(a-teb-bc\right)}^{2}[(\phi +1){\left(ebt+\lambda \right)}^{2}+2Mb(1+2\theta )(2\phi \theta -2\phi +\theta -2)]}{2{\left[{\left(teb+\lambda \right)}^{2}{\left(\phi +1\right)}^{2}+Mb(2\phi +1)(2\phi \theta -4\phi +\theta -4)\right]}^{2}}+Gt$$ (43)

$$\pi_{r}^{CF}=\frac{-M^{2}b(\phi +1)(2\phi +1){\left(a-teb-bc\right)}^{2}[(2\phi^{2}\theta -4\phi^{2}+\phi \theta -5\phi -1)]}{{\left[{\left(teb+\lambda \right)}^{2}{\left(\phi +1\right)}^{2}+Mb(2\phi +1)(2\phi \theta -4\phi +\theta -4)\right]}^{2}}$$ (44)

$$\pi_{c}^{CF}=\frac{-M(\phi +1){\left(a-teb-bc\right)}^{2}[{\left(teb+\lambda \right)}^{2}{\left(\phi +1\right)}^{3}+2Mb{\left(2\phi +1\right)}^{2}(2\phi \theta -3\phi +\theta -3)]}{2{\left[{\left(teb+\lambda \right)}^{2}{\left(\phi +1\right)}^{2}+Mb(2\phi +1)(2\phi \theta -4\phi +\theta -4)\right]}^{2}}+Gt$$ (45)

$$U_{m}^{CF}=\frac{-M{\left(\varphi +1\right)}^{2}{\left(a-teb-bc\right)}^{2}}{2[{\left(teb+\lambda \right)}^{2}{\left(\varphi +1\right)}^{2}+Mb(2\varphi +1)(2\varphi \theta -4\varphi +\theta -4)]}+Gt$$ (46)

$$U_{r}^{CF}=\frac{-M{\left(\phi +1\right)}^{3}{\left(a-teb-bc\right)}^{2}[\phi {\left(teb+\lambda \right)}^{2}(\phi +1)+2Mb{\left(2\phi +1\right)}^{2}]}{2{\left[{\left(teb+\lambda \right)}^{2}{\left(\phi +1\right)}^{2}+Mb(2\phi +1)(2\phi \theta -4\phi +\theta -4)\right]}^{2}}+\phi Gt$$ (47)

$$CS^{CF}=\frac{M^{2}b{\left(2\varphi +1\right)}^{2}{\left(\varphi +1\right)}^{2}{\left(a-teb-bc\right)}^{2}}{2{\left[{\left(teb+\lambda \right)}^{2}{\left(\varphi +1\right)}^{2}+Mb(2\varphi +1)(2\varphi \theta -4\varphi +\theta -4)\right]}^{2}}$$ (48)

$$W^{CF}=\frac{-M(\phi +1){\left(a-teb-bc\right)}^{2}[{\left(teb+\lambda \right)}^{2}{\left(\phi +1\right)}^{3}+Mb{\left(2\phi +1\right)}^{2}(4\phi \theta -7\phi +2\theta -7)]}{2{\left[{\left(teb+\lambda \right)}^{2}{\left(\phi +1\right)}^{2}+Mb(2\phi +1)(2\phi \theta -4\phi +\theta -4)\right]}^{2}}+Gt$$ (49)

## 4.Comparison and analysis

**Theorem 1:** ∂*β*^*NF*^/∂*λ*>0, ∂*Q*^*NV*^/∂*λ*>0, $\partial \pi_{m}^{NF}/\partial \lambda >0$, $\partial \pi_{r}^{NF}/\partial \lambda >0$, $\partial CS^{NF}/\partial \lambda >0$.

See Appendix A in S1 Appendix for proof.

Theorem 1 shows that with the increase of low-carbon preference *λ* of consumers, the low carbon level *β* decided by manufacturers and the order quantity *Q* decided by retailers, the profit *π*_*m*_ obtained by manufacturers, the profit *π*_*r*_ obtained by retailers, the overall profit of the supply chain and the consumer surplus *CS* will all increase accordingly, and since *W* = *π*_*c*_+*CS*, the social welfare will also increase.

**Theorem 2:** ∂*β*^*NF*^/∂*M*>0, ∂*Q*^*NF*^/∂*M*>0, $\partial \pi_{m}^{NF}/\partial M>0$, $\partial \pi_{r}^{NF}/\partial M>0$, ∂*CS*^*NF*^/∂*M*>0.

See Appendix B in S1 Appendix for proof.

Theorem 2 shows that with the increase of low carbon cost coefficient *M*, the low carbon level *β* determined by the manufacturer and the order quantity *Q* determined by the retailer, the profit *π*_*m*_ obtained by the manufacturer, the profit *π*_*r*_ obtained by the retailer, the overall profit of the supply chain and the consumer surplus *CS* all decrease. At the same time, since *W* = *π*_*c*_+*CS*, social welfare will also decrease, which indicates that the greater the cost factor *M* of carbon emission reduction, the more unfavorable it will be to the overall supply chain.

**Theorem 3:** ∂*β*^*NF*^/∂*ϕ*<0, ∂*Q*^*NF*^/∂*ϕ*<0, $\partial \pi_{m}^{NF}/\partial \phi <0$, $\partial \pi_{c}^{NF}/\partial \phi <0$, ∂*CS*^*NF*^/∂*ϕ*<0.

See Appendix C in S1 Appendix for proof.

Theorem 3 shows that in the absence of CSR, the retailer’s fair concern behavior has a negative effect on the supply chain system. In this case, the low carbon level *β* decided by the manufacturer, the order quantity *Q* decided by the retailer, the profit *π*_*m*_ obtained by the manufacturer, the overall profit *π*_*c*_ of the supply chain, the consumer surplus *CS* and the social welfare *W* are all negatively correlated with the fairness concern coefficient *ϕ*.

**Theorem 4:** ∂*β*^*CA*^/∂*θ*>0, ∂*Q*^*CA*^/∂*θ*>0, $\partial \pi_{m}^{CA}/\partial \theta <0$, $\partial \pi_{r}^{CA}/\partial \theta >0$, $\partial \pi_{c}^{CA}/\partial \theta >0$, ∂*CS*^*CA*^/∂*θ*>0, $\partial U_{m}^{CA}/\partial \theta >0$.

See Appendix D in S1 Appendix for proof.

Theorem 4 shows that when manufacturers consider CSR behavior, although their own profits suffer, the strategy is beneficial to the economic and environmental sustainability of the partners and supply chain system. Therefore, in order to further incentivize manufacturers to take the initiative to assume more social responsibilities, retailers can adopt cost-sharing strategies to reduce manufacturers’ concerns about adding additional costs due to emission reduction investments.

**Theorem 5:** *β*^*NF*^<*β*^*NA*^<*β*^*CF*^<*β*^*CA*^.

See Appendix E in S1 Appendix for proof.

Theorem 5 shows that the CSR behavior of manufacturers is conducive to the low-carbon development of enterprises, while the fair concern behavior of retailers will reduce the carbon emission reduction rate. When manufacturers consider CSR behavior and retailers show a preference for fairness concerns, carbon reduction rates are higher than when supply chain members do nothing. In addition, from the perspective of carbon emission reduction rate alone, the emission reduction rate is highest when the manufacturer has CSR behavior and the retailer makes fair and neutral decisions.

**Theorem 6:** *Q*^*NF*^<*Q*^*NA*^<*Q*^*CA*^, and when *Mb*+(*ϕ*+1)[4*Mbθ*−(*bet*+*λ*)^2^]>0, *Q*^*NF*^<*Q*^*NA*^<*Q*^*CA*^<*Q*^*CF*^. Conversely, *Q*^*NF*^<*Q*^*CF*^<*Q*^*NA*^<*Q*^*CA*^.

See Appendix F in S1 Appendix for proof.

Theorem 6 shows that the CSR behavior of the manufacturer is conducive to expanding the market share of the product, while the fair concern behavior of the retailer will reduce the order quantity of the product. For the product market demand, if *Mb*+(*ϕ*+1)[4*Mbθ*−(*bet*+*λ*)^2^]>0, the manufacturer has CSR behavior and the retailer considers the fairness concern preference decision, which indicates that when consumers have low preference for the product, the retailer’s fairness concern behavior will also increase the market demand for the product. On the contrary, if *Mb*+(*ϕ*+1)[4*Mbθ*−(*bet*+*λ*)^2^]<0, when the manufacturer has CSR behavior and the retailer makes fair neutral decision, the market demand is the largest, indicating that when the consumer has a high preference for low-carbon products, the manufacturer considers CSR to increase the product sales more than the retailer’s fair concern behavior.

**Theorem 7:** *ω*^*NA*^>*ω*^*NF*^>*ω*^*CF*^, *ω*^*NA*^>*ω*^*CA*^.

See Appendix F in S1 Appendix for proof.

Theorem 7 states that both the CSR behavior of the manufacturer and the fair concern behavior of the retailer will reduce the wholesale price of the product. At the same time, compared with the single behavior influence, the wholesale price of the product will further decrease when the two behaviors exist simultaneously. This shows that manufacturers adopt the strategy of reducing wholesale prices to increase product orders, so as to make up for the cost of CSR input, while retailers pursue fair profit distribution through lower wholesale prices.

**Theorem 8:** When *λ*<*teb*/3, *P*^*NA*^>*P*^*NF*^>*P*^*CA*^.

See Appendix F in S1 Appendix for proof.

Theorem 8 shows that when consumers have a low preference for low-carbon products, the CSR behavior of manufacturers and the fair concern behavior of retailers will both reduce the selling price of products, which is obviously consistent with the change trend of wholesale prices. In addition, manufacturers’ CSR behavior has a greater impact on the selling price of products than retailers’ fair concern behavior.

## 5. Analysis of numerical examples

With reference to Cheng et al. (2023) [10], the impacts of CSR behavior and fairness preference on pricing and emission reduction decisions of LCSC are discussed through numerical analysis. The related parameters are described as follows: *a* = 450, *b* = 5, *c* = 4, *M* = 500, *e* = 2, *λ* = 1, *G* = 1200. In addition, combined with the current trading price data of China’s carbon market, the carbon trading price of various places ranges from 0.031 to 0.125 yuan/kg. In order to facilitate statistics and analysis, the unit carbon trading price *t* = 1 is taken. Meanwhile,

in order to verify the feasibility of the established decision model, *θ*,*ϕ*{0,2/3} is taken and other parameters are substituted into different decision modes. The optimal parameter values under different decision modes can be obtained, as shown in Table 1.

**Table 1:** Comparative analysis of parameters in different decision modes.

Variables	Neither is considered *θ* = 0,*ϕ* = 0	Consider only CSR *θ* = 2/3,*ϕ* = 0	Consider only equity concerns *θ* = 0,*ϕ* = 2/3	Considering both *θ* = 2/3,*ϕ* = 2/30
*β*	0.47	0.56	0.33	0.44
*ω*	47.58	38.97	35.6	26.24
*Q*	106.29	127.86	105.92	138.52
*P*	68.84	64.54	68.88	62.38
*π*_*m*_	5664.01	5480.15	4377.46	4076.39
*π*_*r*_	2259.35	3269.46	3525.68	5006.96
*π*_*c*_	7923.56	8749.61	7903.14	9083.35
*CS*	1129.67	1347.40	1121.81	1918.73
*U*_*m*_	5664.01	6569.97	5499.27	5995.12
*U*_*r*_	2259.35	3269.97	2957.83	5627.34

As can be seen from Table 1, CSR behavior of manufacturers and fair concern behavior of retailers have an impact on pricing and emission reduction decisions of LCSC. Among them, although the CSR behavior of manufacturers reduces their own profits, it is conducive to the long-term development of the entire LCSC system. However, retailers’ fairness concerns increase their own profits, but reduce the efficiency of the entire LCSC system. In addition, when the manufacturer has CSR behavior, the retailer’s appropriate consideration of fairness concerns is conducive to improving the overall efficiency of the supply chain.

### 5.1 The impact of CSR and fairness concerns on emission reduction and pricing decisions

**Fig 1:**
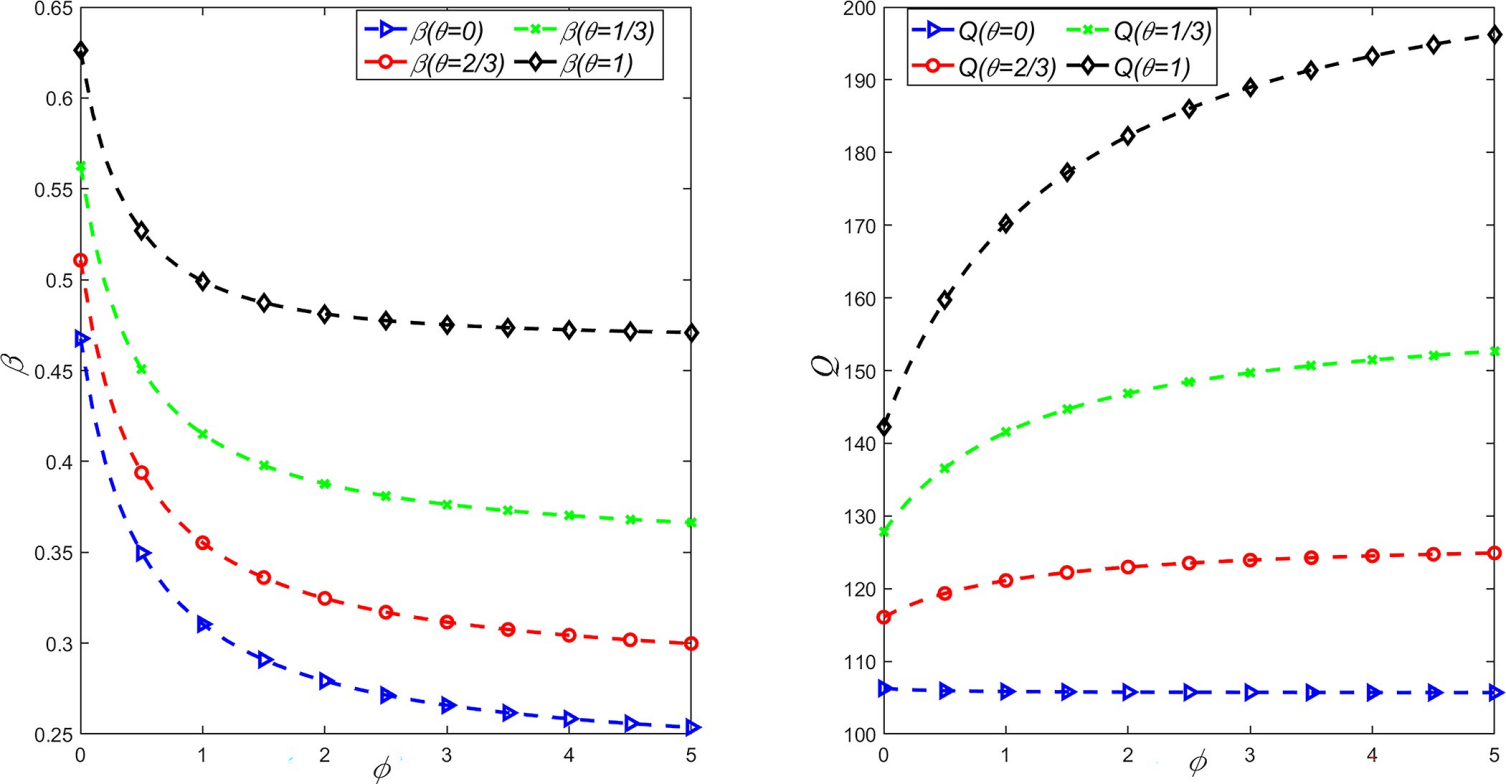
The influence of *θ* and *φ* on emission reduction rate *β* and order quantity *Q*.

**Fig 2:**
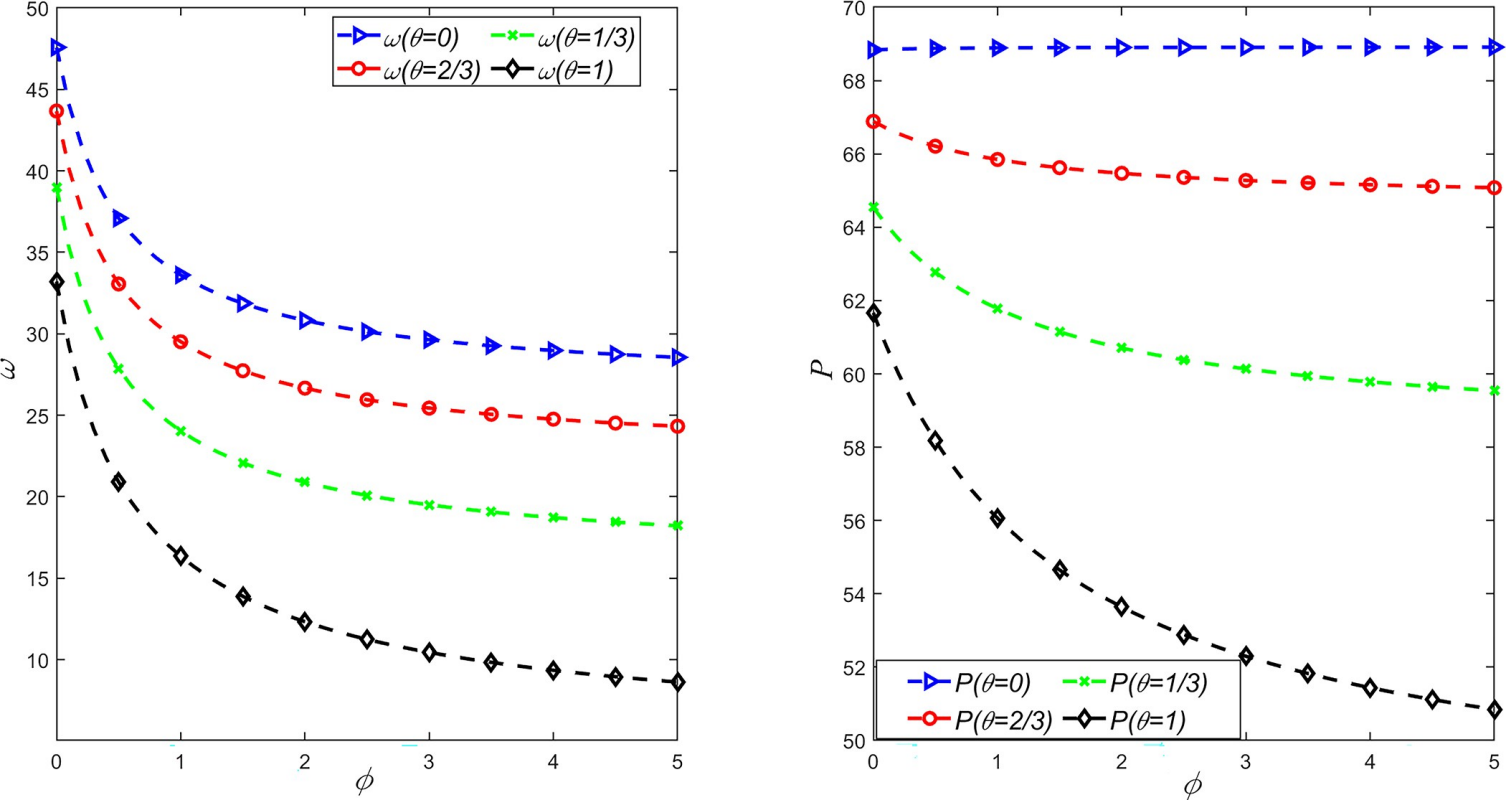
The influence of *θ* and *φ* on wholesale price *ω* and retail price *P*.

In order to study the influence of fairness concern coefficient and CSR on emission reduction rate, order volume, wholesale price and retail price, *θ* = {0,1/3,2/3,1} is taken, and the above parameters are substituted into the expression of decision variables under different situations. It can be obtained that the relationship between emission reduction rate *β* and market demand *Q* and fairness concern coefficient *ϕ* and CSR behavior coefficient *θ* is shown in Fig 1, and the relationship between wholesale price *w* and retail price *P* and fairness concern coefficient *ϕ* and CSR behavior coefficient *θ* is shown in Fig 2.

As can be seen from Figs 1 and 2, with the increase of the level of fairness concern of retailers, the carbon emission reduction rate, wholesale price and retail price will decrease, and only when the manufacturer has CSR behavior, the market demand and retail price will have significant changes. At the same time, with the increase of CSR degree of manufacturers, carbon emission reduction rate and market demand continue to increase, while wholesale prices and retail prices gradually decrease. This shows that when the manufacturer has no CSR behavior and the retailer has fairness concerns, the retailer can only reduce the manufacturer’s wholesale price through bargaining to obtain more profits. Obviously, this behavior will increase internal competition between manufacturers and retailers, thereby reducing the carbon reduction rate of the supply chain system. In addition, when manufacturers consider CSR behavior and retailers have an appropriate level of fairness concern, market demand and carbon reduction rates are greater than when manufacturers have no CSR but retailers have fairness concern. Therefore, in order to achieve the sustainable development of a LCSC, partners can adopt cost sharing or the government can consider carbon subsidies to enhance the enthusiasm of manufacturers for CSR input, and retailers’ fair concerns should also be controlled within a reasonable range.

### 5.2 The impact of CSR and fairness concerns on the profits and utility of member enterprises

**Fig 3:**
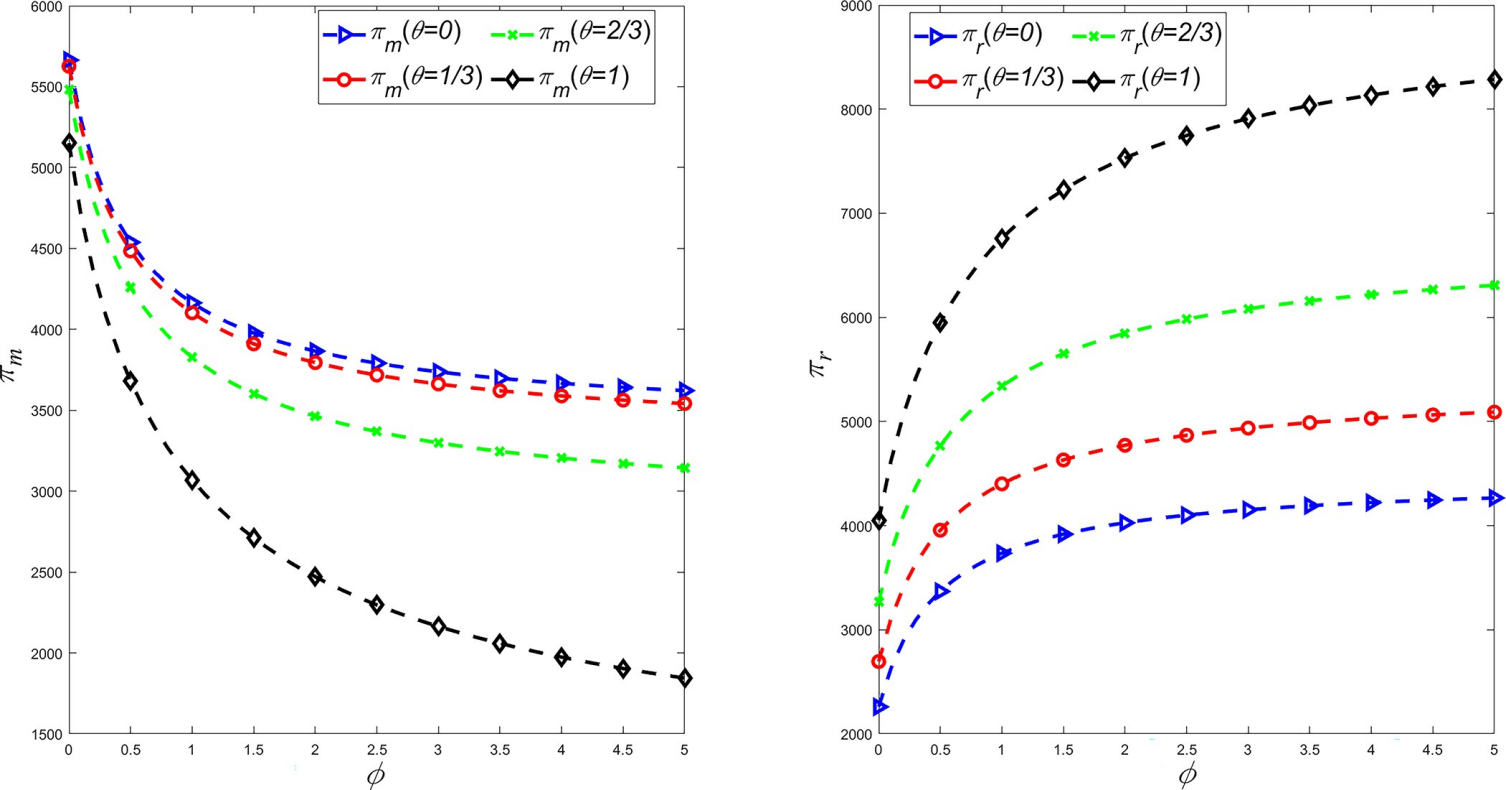
The influence of *θ* and *φ* on manufacturer’s profit *π*_*m*_ and retailer’s profit *π*_*r*_.

**Fig 4:**
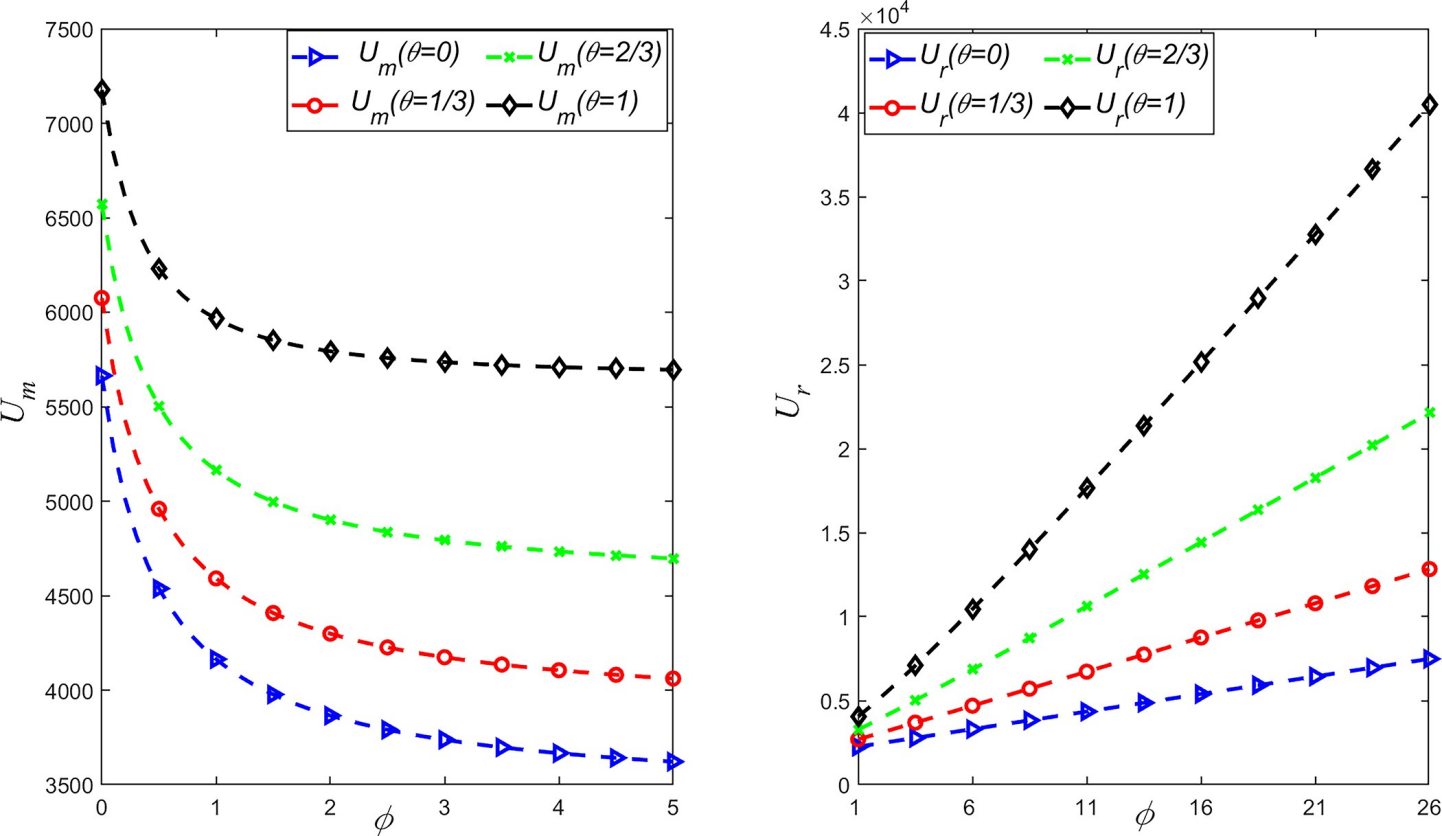
Influence of *θ* and *φ* on manufacturer’s utility *U*_*m*_ and retailer’s utility *U*_*r*_.

To study the influence of fairness concern coefficient and CSR on the profit and utility of supply chain members, *θ* = {0,1/3,2/3,1} is taken and the above parameters are substituted into the expression of decision variables in different situations. The relationship between manufacturer’s profit *π*_*m*_ and retailer’s profit *π*_*r*_ with fairness concern coefficient *ϕ* and CSR behavior coefficient *θ* is shown in Fig 3, and the relationship between manufacturer’s utility *U*_*m*_ and retail utility *U*_*r*_ with fairness concern coefficient *ϕ* and CSR behavior coefficient *θ* is shown in Fig 4.

It can be seen from Figs 3 and 4 that retailers’ profits and utility are positively correlated with their own fair concern behavior, while manufacturers’ profits and utility are negatively correlated with retailers’ fair concern behavior. Meanwhile, manufacturers’ profits are negatively correlated with their own CSR behaviors, while retailers’ profits and utility are positively correlated with manufacturers’ CSR behaviors. Therefore, the behavior of manufacturers to invest extra costs in CSR reduces their own profits, but increases their own utility. However, retailers pay more attention to their own profits and utility due to fairness concerns. When manufacturers invest in CSR, retailers can gain more profit and utility from fairness concerns. And when retailers’fairness concerns are within reasonable bounds, manufacturers’ utility and profit declines are smaller. Therefore, in order to gain more profits and utility, retailers can encourage manufacturers to make CSR investments by sharing the cost of emission reduction. Meanwhile, in order to prevent retailers’ greedy behavior, manufacturers should also restrict retailers’ fair preference behavior.

### 5.3 The impact of CSR and fairness concerns on the overall profit of the supply chain and consumer surplus

**Fig 5:**
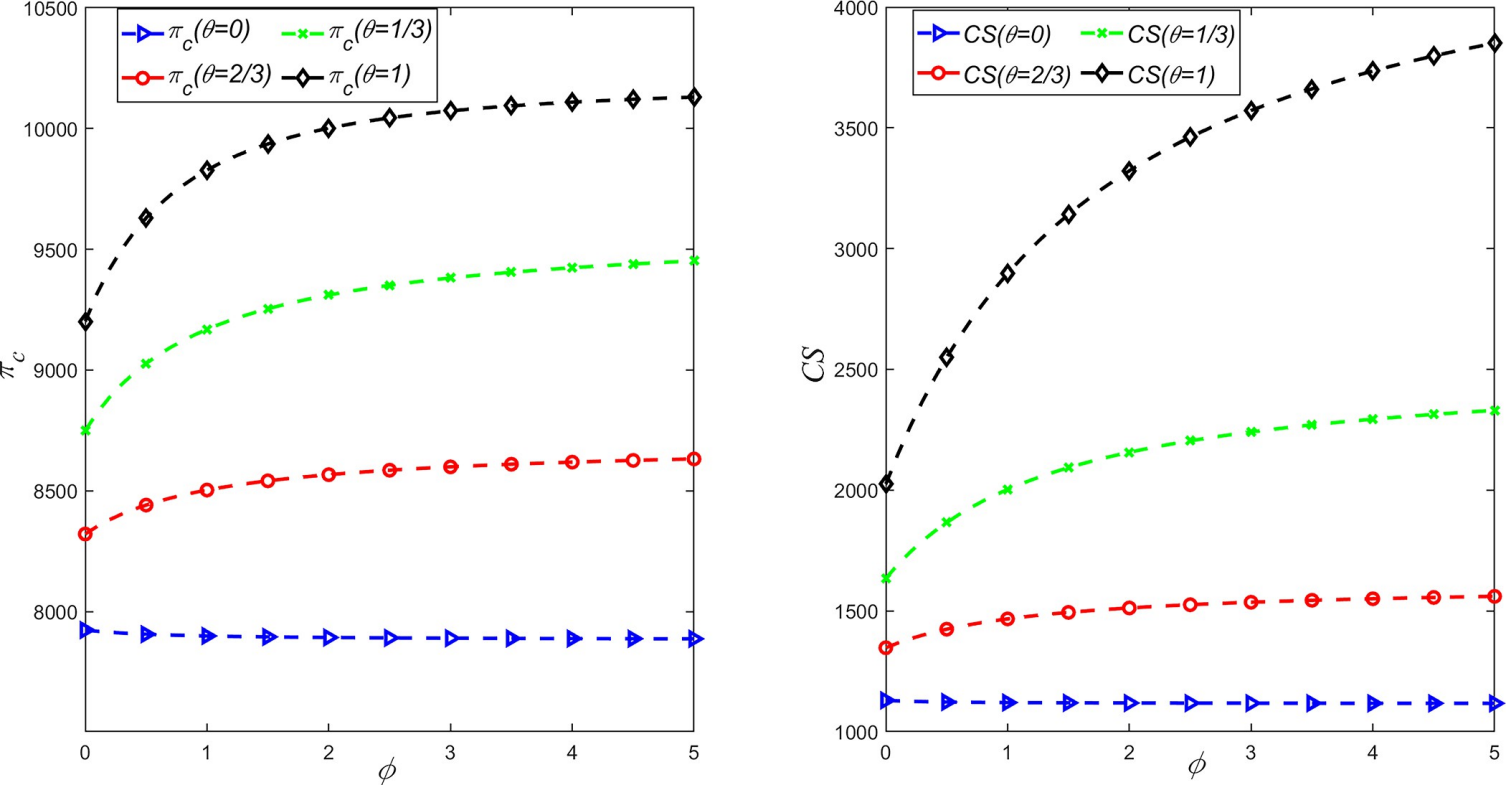
The influence of *θ* and *ϕ* on the overall profit *π*_*c*_ and consumer surplus *CS* of the supply chain.

In order to study the influence of fairness concern coefficient and CSR on the overall profit and consumer surplus of LCSC, *θ* = {0,1/3,2/3,1} is taken, and the above parameters are substituted into the expression of decision variables under different situations. It can be obtained that the relationship between the overall profit *π*_*c*_ of the supply chain and consumer surplus *CS*, the fairness concern coefficient *ϕ* and the CSR behavior coefficient *θ* is shown in Fig 5.

As can be seen from Fig 5, when the manufacturer does not consider CSR behavior, both the overall profit and consumer surplus of the LCSC are negatively correlated with the fairness concern coefficient of retailers. However, when the manufacturer invests in CSR, the relationship among the three becomes positively correlated, and with the increase of CSR behavior coefficient *θ*, the retailer’s fair concern behavior will have a greater positive effect on the overall profits of the LCSC and the consumer surplus. Therefore, only when manufacturers sacrifice part of their own profits to increase CSR input, retailers’ fair concern behavior will have a positive impact on themselves, the LCSC as a whole, and social welfare. However, in the actual LCSC operation management, manufacturers will not actively choose to sacrifice their own profits to obtain the optimal profit of the entire system. When making decisions on LCSC management, retailers should take the initiative to cooperate with manufacturers and share part of the investment cost, so as to realize the sustainable development of the economy and environment of the LCSC system.

## 6. Conclusion

In the context of carbon trading, this paper studies CSR and fairness concerns in manufacturer-led LCSC by using backward derivation and Stackelberg game theory. Overall, the relationship between the various parameters and the overall profit and utility was comprehensively compared and analyzed through four scenarios of retailer fairness concerns and fairness neutrality with and without manufacturer CSR involvement. On this basis, this paper focuses on analyzing the influence of CSR and fairness concern behavior on the whole low carbon supply chain and the profits and utility of each member, and further gives decision-making suggestions on CSR and fairness concern behavior in low carbon supply chain.

The results show that if consumers’ preference for low-carbon products is improved, it will not only promote the manufacturers to improve the emission reduction level, but also increase the profits of the member enterprises of the LCSC, while the carbon emission reduction cost coefficient shows the opposite effect. This shows that in the decision-making process of LCSC operation based on the background of carbon trading, retail enterprises, as a party in direct contact with consumers, should publicize the low-carbon characteristics of products more to enhance consumers’ low-carbon preference, and then obtain more product order quantity, and finally realize the profit and utility growth of manufacturers and retailers. Meanwhile, manufacturing enterprises should appropriately improve the level of carbon emission reduction technology, which is not only conducive to improving the competitiveness of their own products, but also conducive to promoting the overall development of LCSC by promoting the change of consumer behavior. In addition, our research also found that regardless of whether retailers have fair concern behavior, the increase in CSR input of manufacturers can help improve emission reduction, supply chain system efficiency, and social welfare, but is not conducive to the growth of their own profits. On the other hand, retailers’ equity-conscious behavior increases their own profits, but reduces the level of carbon reduction in the system and the profits of their partners.

The research of this paper is of great significance to guide the sustainable development of LCSC. On the one hand, although manufacturers’ investment in CSR is beneficial to the sustainable development of the economy and environment of the supply chain system, they suffer losses in profits as a result.This not only dampens the enthusiasm of manufacturers to take the initiative in CSR investment but is also not conducive to the long-term development of the supply chain system.Therefore, in order to encourage manufacturers to actively invest in CSR behaviors, the government can implement certain subsidy policies for manufacturers to improve their emission reduction enthusiasm and CSR level.On the other hand, retailers will increase their earnings due to fairness concerns. However, this way of increasing profits at the expense of the interests of collaborators will dampen the enthusiasm of manufacturers.To ensure the durability of the cooperation between retailers and manufacturers and actively and effectively carry out carbon emission reduction, a cost-sharing contract can be established between the two to increase the earnings of both parties while reducing the total carbon emissions of the system. This paper helps to understand the relationship between fairness concerns, CSR, carbon reduction rate, price and profit, and helps corporate decision makers to choose effective price strategies and emission reduction strategies, and guides enterprises to make CSR investment and behavior decisions.

It should be noted that the study has some limitations. First of all, this paper only considers a single two-level LCSC, but the actual LCSC is often more complex. Secondly, the demand function in this paper does not consider the relevant influence of CSR and fairness concern behavior on LCSC decision-making under carbon trading in a random environment. These questions provide the direction for our future research.

## Supporting information

S1 Appendix (DOCX)
