# Supplementary material for: A low-carbon supply chain pricing mechanism considering CSR under carbon cap-and-trade policy
Source: PLoS One. 2024 Oct 25;19(10):e0311913. doi: 10.1371/journal.pone.0311913 (PMC11508159; doi:10.1371/journal.pone.0311913)
Supplement: S1 Appendix — (DOCX) [file pone.0311913.s001.docx]

**Appendix A**

**Prove proposition 1.** The derivative of $\beta^{NF}$, $Q^{NF}$, $\pi_{m}^{NF}$, $\pi_{r}^{NF}$ and $CS^{NF}$ with respect to $\lambda$ is:,,,,.

Because $Q$>0, $a-bet-bc>0$, and From 3.2.2 we know that, we can easily know that: $\partial\beta^{NF}/\partial\lambda>0$, $\partial Q^{NF}/\partial\lambda>0$, $\partial\pi_{m}^{NF}/\partial\lambda>0$, $\partial\pi_{r}^{NF}/\partial\lambda>0$, $\partial CS^{NF}/\partial\lambda>0$.

**Appendix B**

**Prove proposition 2.** The derivative of $\beta^{NF}$, $Q^{NF}$, $\pi_{m}^{NF}$, $\pi_{r}^{NF}$ and $CS^{NF}$with respect to $M$ is: ,

,

,

,

.

Similar to proposition 1, Because $a-bet-bc>0$, , we can easily know that $\partial\beta^{NF}/\partial M>0$, $\partial Q^{NF}/\partial M>0$, $\partial\pi_{m}^{NF}/\partial M>0$,$\partial\pi_{r}^{NF}/\partial M>0$, $\partial CS^{NF}/\partial M>0$.

**Appendix C**

**Prove proposition 3.** The derivative of $\beta^{NF}$, $Q^{NF}$, $\pi_{m}^{NF}$, $\pi_{c}^{NF}$ and $CS^{NF}$ with respect to is: ,

, ,,.

Similar to proposition 2, we can easily know that: , ,, , .

**Appendix D**

**Prove proposition 4.** The derivative of $\beta^{CA}$, $Q^{CA}$, $\pi_{m}^{CA}$, $\pi_{r}^{CA}$, $\pi_{c}^{CA}$, $CS^{CA}$and $U_{m}^{CA}$ with respect to $\theta$ is: $\frac{\partial\beta^{CA}}{\partial\theta}=\frac{Mb(bet+\lambda)(a-bet-bc)}{[Mb(\theta-4)+(bet+\lambda)^{2}]^{2}}$,$\frac{\partial Q^{CA}}{\partial\theta}=\frac{M^{2}b^{2}(a-bet-bc)}{[Mb(\theta-4)+(bet+\lambda)^{2}]^{2}}$,$\frac{\partial\pi_{m}^{CA}}{\partial\theta}=\frac{M^{3}b^{2}\theta(a-bet-bc)^{2}}{[Mb(\theta-4)+(bet+\lambda)^{2}]^{3}}$,$\frac{\partial\pi_{r}^{CA}}{\partial\theta}=-\frac{2M^{3}b^{2}(a-bet-bc)^{2}}{[Mb(\theta-4)+(bet+\lambda)^{2}]^{3}}$, $\frac{\partial\pi_{c}^{CA}}{\partial\theta}=\frac{M^{3}b^{2}(a-bet-bc)^{2}(\theta-2)}{[Mb(\theta-4)+(bet+\lambda)^{2}]^{3}}$,$\frac{\partial CS^{CA}}{\partial\theta}=-\frac{M^{3}b^{2}(a-bet-bc)^{2}}{[Mb(\theta-4)+(bet+\lambda)^{2}]^{3}}$, $\frac{\partial U_{m}^{CA}}{\partial\theta}=\frac{M^{2}b(a-bet-bc)^{2}}{2[Mb(\theta-4)+(bet+\lambda)^{2}]^{2}}$.

From 3.2.2 we know that $Mb(\theta-4)+(bet+\lambda)^{2}<0$, so $\partial\beta^{CA}/\partial\theta>0$, $\partial Q^{CA}/\partial\theta>0$,$\partial\pi_{m}^{CA}/\partial\theta<0$, $\partial\pi_{r}^{CA}/\partial\theta>0$, $\partial\pi_{c}^{CA}/\partial\theta>0$, $\partial CS^{CA}/\partial\theta>0$, $\partial U_{m}^{CA}/\partial\theta>0$.

**Appendix E**

**Prove proposition 5.** By comparing the sizes of $\beta^{NF}$, $\beta^{NA}$, $\beta^{CF}$and $\beta^{CA}$, we can get:

,

,.

Because $4bM-(\lambda+teb)^{2}>0$,, $Mb(\theta-4)+(bet+\lambda)^{2}<0$,

, we can find：$\beta^{NF}<\beta^{NA}<\beta^{CF}<\beta^{CA}$.

**Appendix F**

**Prove proposition 6.** By comparing the sizes of $Q^{NF}$, $Q^{NA}$, $Q^{CF}$ and $Q^{CA}$, we can get:

,

$Q^{CA}-Q^{NA}=\frac{M^{2}b^{2}\theta(a-bet-bc)}{[Mb(\theta-4)+(bet+\lambda)^{2}][4Mb-(bet+\lambda)^{2}]}$,

,,

,

Because $4bM-(\lambda+teb)^{2}>0$,,

$Mb(\theta-4)+(bet+\lambda)^{2}<0$, , we can get $Q^{NF}<Q^{NA}<Q^{CA}$. If ,it is easy to know:$Q^{NF}<Q^{NA}<Q^{CA}<Q^{CF}$. Otherwise, we can get$Q^{NF}<Q^{CF}<Q^{NA}<Q^{CA}$.

**Appendix G**

**Prove proposition 7.** By comparing the sizes of $\omega^{NA}$, $\omega^{NF}$, $\omega^{CF}$ and $\omega^{CA}$, we can get:,

$\omega^{NA}-\omega^{CA}=\frac{M\theta(a-bet-bc)(2Mb-\lambda^{2}-teb\lambda)}{[Mb(4-\theta)-(bet+\lambda)^{2}][4Mb-(bet+\lambda)^{2}]}$,

.

Because $2bM-(\lambda+teb)^{2}>0$, $Mb(\theta-4)+(bet+\lambda)^{2}<0$,$0<\theta<1$, it is easy to know $\omega^{NA}>\omega^{NF}$, $\omega^{NA}>\omega^{CA}$, $\omega^{NF}>\omega^{CF}$, we can get $\omega^{NA}>\omega^{NF}>\omega^{CF}$.

**Appendix H**

**Prove proposition 8.** By comparing the sizes of $P^{NA}$, $P^{NF}$和$P^{CA}$, we can get:

,

$P^{NA}-P^{CA}=\frac{-M\theta(a-bet-bc)(Mb-teb\lambda-\lambda^{2})}{[Mb(\theta-4)+(bet+\lambda)^{2}][4Mb-(bet+\lambda)^{2}]}$,

.

When $\lambda<teb/3$, because $2bM-(\lambda+teb)^{2}>0$, $a-bet-bc>0$, we can get$P^{NA}-P^{NF}>0$,$P^{NA}-P^{CA}>0$. Let , take the derivative of $\lambda$ and find $\partial F(\lambda)/\partial\lambda<0$, and because $F(\lambda\text{=}teb/3)>0$, when $\lambda<teb/3$, $P^{NF}-P^{CA}>0$.
